# Evidence for horizontal transfer of a secondary metabolite gene cluster between fungi

**DOI:** 10.1186/gb-2008-9-1-r18

**Published:** 2008-01-24

**Authors:** Nora Khaldi, Jérôme Collemare, Marc-Henri Lebrun, Kenneth H Wolfe

**Affiliations:** 1 Smurfit Institute of Genetics, Trinity College Dublin, Dublin 2, Ireland; 2 2UMR5240 CNRS/UCB/INSA/BCS, Bayer Cropscience, 69263 Lyon cedex 09, France

## Abstract

**Background:**

Filamentous fungi synthesize many secondary metabolites and are rich in genes encoding proteins involved in their biosynthesis. Genes from the same pathway are often clustered and co-expressed in particular conditions. Such secondary metabolism gene clusters evolve rapidly through multiple rearrangements, duplications and losses. It has long been suspected that clusters can be transferred horizontally between species, but few concrete examples have been described so far.

**Results:**

In the rice blast fungus *Magnaporthe grisea*, the avirulence gene *ACE1 *that codes for a hybrid polyketide synthase-nonribosomal peptide synthetase (PKS-NRPS) belongs to a cluster of 15 genes involved in secondary metabolism. Additional related clusters were detected in the ascomycetes *Chaetomium globosum*, *Stagonospora nodorum *and *Aspergillus clavatus*. Gene-by-gene phylogenetic analysis showed that in *C. globosum *and *M. grisea*, the evolution of these *ACE1*-like clusters is characterized by successive complex duplication events including tandem duplication within the *M. grisea *cluster. The phylogenetic trees also present evidence that at least five of the six genes in the homologous *ACE1 *gene cluster in *A. clavatus *originated by horizontal transfer from a donor closely related to *M. grisea*.

**Conclusion:**

The *ACE1 *cluster originally identified in *M. grisea *is shared by only few fungal species. Its sporadic distribution within euascomycetes is mainly explained by multiple events of duplication and losses. However, because *A. clavatus *contains an *ACE1 *cluster of only six genes, we propose that horizontal transfer from a relative of *M. grisea *into an ancestor of *A. clavatus *provides a much simpler explanation of the observed data than the alternative of multiple events of duplication and losses of parts of the cluster.

## Abstract 2

Phylogenetic and comparative genomic analysis of orthologs of the Magnaporthe grisea ACE1 cluster reveals evidence for horizontal transfer of part of this cluster from an M. grisea-like ancestor into an ancestor of Aspergillus clavatus.

## Background

In filamentous fungi, genes involved in the same secondary metabolite biosynthetic pathway are often located at the same locus in the genome and co-expressed, defining gene clusters [1]. Genomic clustering of genes with related cellular functions (but unrelated sequences) also occurs in other eukaryotes including mammals, nematodes and plants [2-4]. In mammals, it has been shown that clusters of co-expressed genes tend not to be rearranged among species, which indicates that natural selection can act to conserve gene order [5,6]. Similarly in fungi, natural selection seems to act to conserve gene clusters as exemplified in *Aspergillus *species by the cluster for the biosynthesis of aflatoxin and sterigmatocystin that has been maintained as a cluster, despite many internal rearrangements, for at least 120 million years [7,8]. The evolutionary mechanisms by which these clusters are created and maintained are unclear, but there is evidence that some instances of clustering result from strong natural selection. For example, the *DAL *cluster involved in nitrogen metabolism in *Saccharomyces cerevisiae *was formed relatively recently by a series of near-simultaneous relocations of genes that were previously scattered around the genome [9]. Other mechanisms involved in the formation and maintenance of clusters include selection for co-regulation by chromatin remodelling, epistatic selection for tight linkage between genetically interacting genes, and the "selfish operon" hypothesis of origin by horizontal gene transfer (HGT) [2,10-13]. Indeed, the clustering of the genes from a pathway at a single locus certainly facilitates HGT of genes involved in the same cellular function [10,14], increasing its likelihood.

Despite frequent speculation (reviewed in [15]), and even though some clear examples of HGT of single genes between fungal species [16] or from bacteria to fungi [17] are known, there are few reports that conclusively demonstrate HGT of a fungal secondary metabolite cluster. The strongest candidate reported so far is the epipolythiodioxopiperazine (ETP) synthase gene cluster, recently analyzed by Patron *et al *[18], but even in this instance alternative evolutionary scenarios can be contemplated (see Discussion). One of the best-known cases of possible HGT of a fungal secondary metabolite cluster concerns the fungal β-lactam (penicillin) antibiotic biosynthetic genes of *Penicillium *species. This proposal was originally made when bacterial and fungal isopenicillin-*N*-synthetases were found to have unexpectedly highly similar protein sequences [19-21]. However, subsequent phylogenetic analyses of these proteins failed to provide robust support for their HGT [22,23].

The rice blast fungus *Magnaporthe grisea *is one of the richest known fungi in terms of secondary metabolite gene clusters [24,25]. One of them contains the avirulence gene *ACE1 *that encodes a hybrid polyketide synthase-nonribosomal peptide synthetase (PKS-NRPS) likely involved in the biosynthesis of an avirulence signal recognized by rice cultivars carrying the resistance gene *Pi33 *[26]. The *ACE1 *cluster contains 15 genes that are co-expressed specifically during the appressorium mediated penetration of the fungus into host tissues (Collemare *et al*, unpublished results). During annotation of the *ACE1 *cluster, a similar cluster was identified in the related animal pathogen *Chaetomium globosum*. We were then interested in identifying possible homologous clusters in other fungi in order to decipher its evolutionary history. In the present study, we combine phylogenetics and comparative genomics to identify orthologs of the *M. grisea ACE1 *cluster in other ascomycetes. We define a set of three genes that are shared across all instances of the cluster and hence are probably ancestral to it. This analysis revealed that the cluster in *M. grisea *expanded by internal duplication, and that after this duplication, part of the *ACE1 *cluster was likely horizontally transferred from an *M. grisea*-like ancestor into an ancestor of *Aspergillus clavatus*.

## Results

### Identification of homologous ACE1 clusters in other filamentous fungi

**Figure 1:**
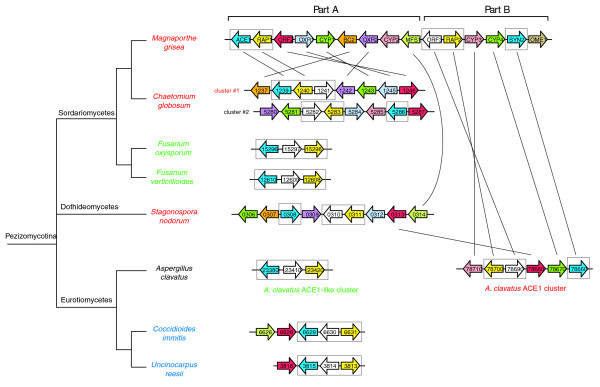
*ACE1 *and *ACE1*-like gene clusters in filamentous fungi. Colors indicate gene orthology in different species and paralogs in the same species. Horizontal lines indicate genes that are adjacent in the genome, with gene orientations as shown. Genomic regions are not drawn to scale. Parts A and B of the *M. grisea *cluster as identified in the text are marked. The core set of three genes inferred to have been present in the ancestral cluster are boxed. Vertical lines indicate the closest relatives of genes in the *M. grisea *cluster and one of the *A. clavatus *clusters, based on phylogenetic analyses (Figure 2 and Additional data file 1). The species phylogeny is based on the whole-genome supertree analysis of Fitzpatrick *et al *[27]; in that study the placement of Dothideomycetes relative to Sordariomycetes and Eurotiomycetes varied depending on the method of analysis, so we have shown it as a trichotomy. The analysis of Hane *et al *of the complete *S. nodorum *genome placed Dothideomycetes and Sordariomycetes in a clade with Eurotiomycetes outside [47]. Species-specific gene nomenclature is shown, except for *M. grisea *(Collemare *et al*, unpublished results). Red, green and blue coloring of species names corresponds to the labelling of individual genes from the clusters in Figure 2 and Additional data file 1.0.

The *ACE1 *secondary metabolism gene cluster of *M. grisea *comprises 15 genes: *ACE1 *and *SYN2 *are PKS-NRPS hybrid genes; *RAP1 *and *RAP2 *code for enoyl reductases; *CYP1-CYP4 *for cytochrome P450 monoxygenases; *ORFZ *for an α/β-hydrolase; *OXR1 *and *OXR2 *for oxidoreductases; *MFS1 *codes for a transporter in the MFS superfamily; *BC2 *codes for a binuclear zinc finger transcription factor; *OME1 *codes for an O-methyl transferase; and *ORF3 *has no homology to known proteins (Collemare *et al*, unpublished results). To find gene clusters homologous to the *ACE1 *cluster in other fungal species, we used an algorithm that searched 26 fungal genomes for loci where at least three likely orthologs of genes from the *ACE1 *cluster were linked (see Materials and methods). This search identified nine similar clusters in seven fungal species from the subphylum Pezizomycotina: three Sordariomycetes (*Chaetomium globosum*, *Fusarium oxysporum *and *F. verticillioides*), one Dothideomycete (*Stagonospora nodorum*) and three Eurotiomycetes (*Aspergillus clavatus*, *Coccidioides immitis *and *Uncinocarpus reesii*) (Figure 1).

Two types of clusters related to the *ACE1 *cluster were identified: large clusters with eight or more genes are found in *M. grisea*, *C. globosum *and *S. nodorum*, whereas smaller clusters with three to six genes are found in the three Eurotiomycetes and in *Fusarium *species (Figure 1). *C. globosum *is unusual as its genome contains two large *ACE1*-like clusters, which we refer to as clusters 1 and 2. Similarly, the *A. clavatus *genome has two clusters as discussed below. Interestingly, a core set of three genes (homologs of *ACE1*, *RAP1 *and *ORF3*; boxed in Figure 1) is present in all eight species. The presence of this core suggests that the physical linkage between these three genes is ancient and can be inferred to have existed in the common ancestor of all the genomes considered in Figure 1. As well as the genes in the eight clusters shown in Figure 1, we also identified a small number of single homologs of genes from the *M. grisea ACE1 *cluster that are located at dispersed genomic locations in other species.

### Phylogenetic analysis of the ACE1 cluster in filamentous fungi

**Figure 2:**
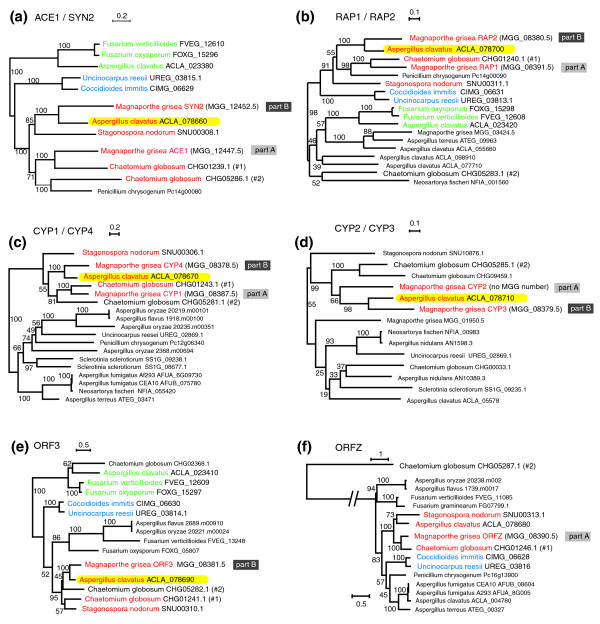
Maximum likelihood trees for *ACE1 *cluster genes and their homologs. (a) *ACE1 *and *SYN2*; (b) *RAP1 *and *RAP2*; (c) *CYP1 *and *CYP4*; (d) *CYP2 *and *CYP3*; (e) *ORF3*; (f) *ORFZ*. In each tree, genes that appear in Figure 1 are named in color or bold black. Yellow highlighting shows the five genes in the *A. clavatus ACE1 *cluster whose closest relatives are genes from part B of the *M. grisea *cluster. Bootstrap percentages are shown for all nodes. Trees were constructed from amino acid sequences as described in Methods using PHYML after alignment with ClustalW and Gblocks filtering. Trees for the other five genes in the *ACE1 *cluster are shown in Additional data file 1. The values of the shape parameter (α) for the gamma distribution were estimated from the data as 1.329, 1.441, 2.476, 2.615, 2.536 and 0.961 for panels a-f, respectively. The proportions of invariant sites are 0.028, 0.035, 0.030, 0.068, 0.000 and 0.000, respectively. The *M. grisea SYN2 *gene corresponds to parts of the automatically-annotated gene models MGG_12452.5 and MGG_12451.5.

Gene-by-gene phylogenetic analyses were carried out to decipher the evolutionary history of the loci using homologs (even at dispersed locations) of genes from *M. grisea ACE1 *cluster (Figure 2 and Additional data file 1). The first trend evident from this phylogenetic analysis is that genes from clusters in Eurotiomycetes and *Fusarium *spp. are distant from those of the *M. grisea*, *C. globosum *and *S. nodorum *clusters. Indeed, genes in clusters from these last three species define clades supported by high bootstrap values (> 91%), to the exclusion of genes from Eurotiomycetes and *Fusarium *species (Figure 2a,b,e,f). Interestingly, genes from one of the two clusters in *A. clavatus *are more closely related to genes in the *M. grisea ACE1 *cluster than to those in *ACE1*-like clusters from other Eurotiomycetes (see below). In view of the gene contents of the clusters and their phylogenetic relationships, we refer to the large clusters in *M. grisea*, *C. globosum*, *S. nodorum *and the larger of the two clusters in *A. clavatus *as "*ACE1 *clusters", and to the smaller clusters in Eurotiomycetes and *Fusarium *spp. as "*ACE1*-like clusters". These two types of cluster have probably had a long history of independent evolution, although they certainly share a common ancestor.

We then focused on the origins of the duplicated genes in the *M. grisea *cluster. Phylogenetic trees show clearly that in *M. grisea RAP2 *is a paralog of *RAP1*, *CYP3 *is a paralog of *CYP2*, *CYP4 *is a paralog of *CYP1*, and *SYN2 *is a paralog of *ACE1 *(Figure 2a-d). Notably, in each of these pairs, one gene is located on the left-hand side of the *M. grisea *cluster and the other is on the right-hand side. Thus the *M. grisea *cluster appears to have undergone partial tandem duplication at some stage during its evolution, although the gene order is not conserved between the two parts. The presence of two *ACE1 *clusters in *C. globosum *is suggestive of a second block-duplication event in this species. However, for most genes present in both *C. globosum ACE1 *clusters, the copy from cluster 1 forms a clade with their *M. grisea *homologs. This close phylogenetic relationship is observed for *ACE1*, *RAP1*, *ORFZ*, *OXR1*, *CYP1*, and *OXR2*. The only exception to this pattern is *M. grisea ORF3*, which is marginally closer to the *C. globosum *cluster 2 gene, but with low bootstrap support (Figure 2 and Additional data file 1). This observation suggests that the duplication that gave rise to the current *C. globosum *clusters 1 and 2 occurred in a common ancestor of *C. globosum *and *M. grisea*, and that the corresponding cluster 2 in *M. grisea *was lost.

On the basis of this analysis, we divided the *M. grisea *cluster into two parts, A and B, so that each of the duplicated genes in *M. grisea *has one copy in part A and one in part B (Figure 1). Part A in *M. grisea *consists of nine genes, all of which have orthologs in one or both of the clusters in its closest relative *C. globosum*. The clusters in other species consist of homologs of genes from *M. grisea *part A, plus one gene from part B (*ORF3*; see Discussion). The order of the part A genes is not conserved among *M. grisea*, *C. globosum *and *S. nodorum*.

Surprisingly, this phylogenetic analysis shows that five of the six genes from part B of the *M. grisea ACE1 *cluster group with genes from the larger of the two clusters in *A. clavatus*, rather than with the genes in the more closely related (Sordariomycete) species *C. globosum*, or with their part A paralogs in *M. grisea*. Bootstrap values for grouping the *M. grisea *part B genes *SYN2*, *RAP2*, *CYP4*, *CYP3 *and *ORF3 *with their *A. clavatus *homologs are 98-100% (Figure 2a-e). The only gene from part B of the *M. grisea *cluster that does not group with *A. clavatus *is *OME1 *(panel e of Additional data file 1), but this is also the only gene whose detected homolog in *A. clavatus *(*ACLA_002520*) is not physically clustered with the others, which calls its orthology into question. The consistency of this phylogenetic result for part B genes, and its disagreement with the expected species relationships, are indicative of HGT between *A. clavatus *and part B of the *M. grisea *cluster. In contrast seven of the nine genes from part A of the *M. grisea *cluster, including *ACE1 *itself, lie at the expected phylogenetic position forming a clade with *C. globosum *(Figure 2 and Additional file 1; the two exceptions are *CYP2*, which is discordant but has a low bootstrap value of 66%, and *MFS1*, which cannot be analyzed because there is no homolog in the *C. globosum *clusters).

For the four panels in Figure 2 that include sequences from other Eurotiomycetes (*C. immitis *and *U. reesii*) as well as *A. clavatus*, we used the likelihood ratio test (LRT) to test whether the topologies shown (Figure 2a,b,e,f) have significantly higher likelihoods than alternative trees where the Eurotiomycetes were constrained to form a monophyletic group. In all four cases the topology shown in Figure 2 is significantly more likely than the tree expected if genes were inherited vertically (p < 0.001 for each).

### Identifying the direction of gene transfer

To determine whether part B of the cluster was transferred from an *M. grisea*-like donor to an ancestor of *A. clavatus*, or vice versa, we examined phylogenetic trees constructed from those genes that have orthologs both in species that are close relatives of *M. grisea *and in species that are closer to *A. clavatus*. We would predict that if an ancestor of *A. clavatus *was the recipient of HGT, then the genes in its *ACE1 *cluster would not show the expected close relationship to other Eurotiomycete species such as *C. immitis *and *U. reesii *(Figure 1), and would instead form a clade with the donor lineage (represented by *M. grisea*). Conversely, if the direction of transfer was from an *A. clavatus*-like donor into the *M. grisea *lineage, we would expect the *M. grisea *part B genes not to form a monophyletic clade with the other Sordariomycete species *C. globosum*, and instead to group with *A clavatus*.

In the phylogenetic tree of *ORF3 *sequences, the shared *A. clavatus-M. grisea *branch lies within a clade that contains homologs from the two clusters in *C. globosum*, as well as the Dothideomycete *S. nodorum *(Figure 2e). The *ORF3 *orthologs from *C. immitis *and *U. reesii *clearly lie outside this clade with 95% bootstrap support. Similarly, the phylogenetic tree of *RAP1 *and *RAP2 *orthologs (Figure 2b) shows that the shared branch containing the *A. clavatus *gene and the part B *M. grisea *gene (*RAP2*) lies within a larger clade that includes the *C. globosum *and *M. grisea *part A (*RAP1*) orthologs. The homologs from *C. immitis *and *U. reesii *lie outside (91% bootstrap support). Likewise, the phylogenetic tree of the *ACE1-SYN2 *pair (Figure 2a) places the *A. clavatus *sequence within a Sordariomycete/Dothideomycete clade, distant from the other Eurotiomycetes (*C. immitis *and *U. reesii*). These topologies all indicate that an ancestor of *M. grisea *was the donor of the transferred part B genes, and an ancestor of *A. clavatus *was the recipient.

*ORFZ *is the only gene in the *A. clavatus ACE1 *cluster that does not have a homolog in part B of the *M. grisea *cluster. The origin of this gene in *A. clavatus *is not clear. Phylogenetic analysis (Figure 2f) indicates that *A. clavatus ORFZ *does not group with the *C. immitis *and *U. reesii *genes, and this conclusion is supported by the LRT. This result suggests a foreign origin for *A. clavatus ORFZ*, but the absence of a homolog in *M. grisea *part B makes it impossible to test whether this gene has a similar origin to its five neighboring genes in *A. clavatus*.

We conclude that there is phylogenetic support for the hypothesis that at least five of the six genes in the *ACE1 *cluster of *A. clavatus *originated by HGT, and that the most probable single donor is a Sordariomycete ancestor related to *M. grisea*.

## Discussion

### The ACE1 cluster is specific to few fungal species

**Figure 3:**
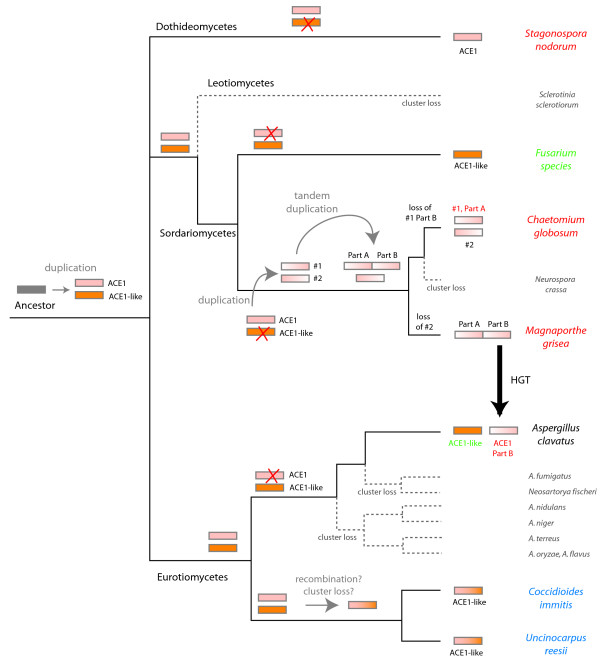
Inferred history of *ACE1 *and *ACE1*-like clusters in filamentous fungi. The gray rectangle corresponds to the ancient core cluster of three genes (*ACE1*, *RAP1*, *ORF3*) that is common to all *ACE1 *clusters (pink) and *ACE1*-like clusters (orange). The black arrow denotes the inferred HGT of part B of the cluster from a donor related to *M. grisea *to the *A. clavatus *recipient. Dashed branches and smaller fonts indicate euascomycetes that were included in our analysis but lack the clusters entirely. Phylogenetic relationships are based on [27] and N Fedorova and N Khaldi, unpublished data, for the topology within the genus *Aspergillus*. The tree is not drawn to scale.

A complete *ACE1 *cluster is present in only four of the 23 sequenced Pezizomycotina genomes (*M. grisea*, *C. globosum*, *S. nodorum *and *A. clavatus*). Such a sporadic distribution could be the result of either independent HGTs or frequent losses of the whole cluster in different lineages (Figure 3). We favor the latter explanation because - with the exception of *A. clavatus *- our phylogenetic trees of genes from the cluster have topologies that are in broad agreement with the expected species phylogeny [27]. We suggest that an *ACE1*-like cluster consisting of at least three genes (homologous to *ACE1*, *RAP1 *and *ORF3*) existed in the common ancestor of Pezizomycotina, but this cluster has been lost in many lineages subsequently. The scheme in Figure 3 identifies four independent lineages (shown by dashed lines) in which all copies of the cluster have been lost. We cannot tell, with current data, whether genes such as *OXR1 *that are present in the *ACE1 *clusters of Sordariomycetes and Dothideomycetes but not in the *ACE1*-like clusters of Eurotiomycetes correspond to lineage-specific additions or losses.

Any tree showing apparent HGT of a gene can also be explained by an alternative scenario of gene duplications and losses. However, the situation reported here is rather different to typical cases of possible HGT of individual genes, because it involves multiple genes that are arranged as a large tandem duplication (in *M. grisea*). The fact that the *A. clavatus ACE1 *cluster forms a clade with the *M. grisea *part B genes (to the exclusion of the part A genes) means that the only alternative scenario to HGT is one where the part A/part B tandem duplication occurred right at the base of the tree in Figure 3. This scenario would then necessitate at least four events of precise loss of exactly one part of the tandemly duplicated set of genes: part B in *C. globosum*, part B in the ancestor of *C. immitis *and *U. reesii*, part B in *S. nodorum*, and part A in *A. clavatus*. Because of the precise nature of the deletion required (and choice of gene copy to delete), we do not regard this scenario as likely.

The discontinuous distribution of the *ACE1 *cluster among fungal species suggests that evolutionary constraints act to maintain this cluster only in few species. As *M. grisea*, *S. nodorum *and *C. globosum *are plant or animal pathogens, it is tempting to speculate that the *ACE1 *cluster is involved in the infection process of these three species. The metabolite produced by this biosynthetic pathway may be an important pathogenicity factor, but such a role remains to be determined. *A. clavatus *is different as it is not pathogenic. The presence of the *ACE1 *cluster in *A. clavatus *may arise from selection involving an unknown biological role of this metabolite in this fungus. Identifying the molecules made by these different clusters will be necessary to understand the role of the *ACE1 *cluster in fungal biology and could give clues about evolution of the ancestral biosynthetic pathway controlled by this cluster.

### ACE1 cluster evolution in Sordariomycetes involved several duplication events

The *ACE1 *cluster has a complex history with multiple events of large-scale duplication and multiple losses. The scenario we infer is summarized in Figure 3. An ancient duplication produced the large *ACE1 *and smaller *ACE1*-like clusters. A second duplication event in an ancestral Sordariomycete gave rise to the two clusters (1 and 2) presently seen in *C. globosum*. This event occurred prior to the speciation between *C. globosum *and *M. grisea*, but *M. grisea *later lost its counterpart of cluster 2. Independently, cluster 1 underwent a tandem duplication event, generating parts A and B. This tandem duplication survived in *M. grisea*, but in *C. globosum *the addition (part B of cluster 1) was lost again. It might seem simpler to suggest that the part A/B tandem duplication was an event that occurred specifically in *M. grisea *after it diverged from *C. globosum*, but we know that this is incorrect because the part B genes from *M. grisea *form outgroups to a clade consisting of *C. globosum *and *M. grisea *part A genes. We can also be sure that the surviving duplications seen in *M. grisea *and *C. globosum *were separate events because of the topology of the phylogenetic trees: if the surviving genes were descended from the same duplication event we would expect that in the *ACE1-SYN2 *tree, for example, *M. grisea ACE1 *and *SYN2 *should each form a separate monophyletic group with one of the *C. globosum *genes, but that is not seen (Figure 2a). Instead we interpret the trees as indicative of two duplications of the whole cluster in a Sordariomycete ancestor of *M. grisea *and *C. globosum*, the first of which was non-tandem and the second of which was tandem. After this tandem duplication, the *M. grisea *lineage lost its ortholog of cluster 2 of *C. globosum*, and the *C. globosum *lineage lost its ortholog of part B of *M. grisea *(Figure 3). This pattern of frequent loss is consistent with the cluster's sporadic distribution in fungi.

*ORF3 *is unusual as it is inferred to have been present in the ancestor of all *ACE1 *and *ACE1*-like clusters, but in *M. grisea *it is not duplicated and it shows phylogenetic affinity to *A. clavatus *rather than to *C. globosum *or *S. nodorum *(Figure 2e). These properties suggest that a homolog of *ORF3 *was lost from part A of the *M. grisea *cluster, after the tandem duplication occurred. Furthermore, we speculate that the location of *ORF3 *on the boundary between parts A and B may indicate that the tandem duplication event visible in *M. grisea *involved a recombination between two copies of this gene.

Gene order and orientation is quite poorly conserved among the *ACE1 *clusters, as is typical of many secondary metabolism gene clusters [7,8,28]. This makes it all the more striking that the duplicated *M. grisea *genes each have one copy in the part A and one copy in part B. Because the tandem duplication that is evident in the *M. grisea *genome is not particularly recent (it predates the *M. grisea*/*C. globosum *speciation), we suggest that some form of selection has acted on gene order in the cluster, preventing intermixing of the two parts. In this context it is notable that recombination seems to be inhibited in the *M. grisea ACE1 *cluster, because it displays a low frequency of targeted gene replacement, even in a *KU80 *null mutant background where homologous recombination rates are increased ([29]; Collemare *et al*, unpublished results). The way that part A and part B genes of the *ACE1 *cluster are distributed among species may indicate that they are involved in the biosynthesis of different molecules. Alternatively, parts A and B of the *ACE1 *cluster may be each involved in the biosynthesis of independent polyketide precursors that are fused into a final complex molecule as observed for lovastatin [25,30,31]. The fact that all 15 genes in the *M. grisea ACE1 *cluster are co-expressed at a very specific stage of the infection process (Collemare *et al*, unpublished results) favors the hypothesis that both part A and part B genes are involved in same biosynthetic pathway. However, gene knockout experiments have shown that two part B genes (*RAP2 *and *SYN2*) are not essential for the avirulence function supported up to now only by the part A gene *ACE1 *(Collemare *et al*, unpublished results). These latter results suggest that part A and part B genes could be involved in the biosynthesis of two different molecules, with only one (*ACE1*, part A pathway) being recognized by resistant rice cultivars. However, these two hypotheses are both plausible, and await the biochemical characterization of the Ace1 metabolite.

### HGT of a fungal secondary metabolism gene cluster

Although the genomics era has uncovered evidence for widespread horizontal gene transfer among prokaryotes [32,33], and from prokaryotes to eukaryotes [17,34-37] or *vice versa *[38,39], relatively few instances of horizontal gene transfer have been documented from one eukaryote to another [40-42]. Among fungi, the best documented is the transfer of a virulence gene from *S. nodorum *to *Pyrenophora tritici-repens*, which occurred only about 70 years ago [16]. In that case, the transferred DNA fragment was about 11 kb in size but contained only one gene. In this study we showed that part B of the *ACE1 *cluster (30 kb in size, containing 5-6 genes) was likely horizontally transferred from a close ancestor of *M. grisea *(a Sordariomycete) into an ancestor of *A. clavatus *(a Eurotiomyete). The mechanism by which HGT might have occurred remains a matter of speculation, but could perhaps have involved hyphal fusion between species, or endocytosis. Our inference of HGT is valid only if the Sordariomycete and Eurotiomycete clades are monophyletic as shown in Figure 1, but their monophyly is supported by several molecular and systematic analyses [27,43-47].

To our knowledge, our study and the recent work of Patron *et al *[18] are the first reported instances of HGT of groups of linked genes involved in the same pathway between eukaryotic species. In both cases these secondary metabolite clusters show a punctate (sporadic) distribution among other species, with an ancestral cluster apparently having been lost by more species than the number that retain it. This pattern of frequent losses of genes and their occasional reacquisition by HGT resembles the pattern of evolution of "dispensable pathway" genes in ascomycete yeasts [48]. Hall and Dietrich [48] noted that genes whose products function in dispensable pathways are one of the few categories of genes in *S. cerevisiae *that are physically organized into gene clusters. They found that the pathway for biotin synthesis was lost in a yeast ancestor and then regained in the *S. cerevisiae *lineage by a combination of HGT from bacteria and gene duplication with neofunctionalization. One possible explanation for this strange pattern of evolution could be that an intermediate in the pathway is toxic [48], although there is no direct experimental evidence of this. If a pathway can confer a selective advantage in some circumstances but also involves the production of a toxic intermediate, there can be strong selection in favor of the pathway in some conditions and strong selection against it in others. The consequences of such a situation could include the formation of physical gene clusters (to reduce the chances of coding for only part of the pathway, or for strong repression of transcription mediated by chromatin remodelling), and occasional selection for re-gain of function by HGT. Further exploration of this hypothesis will require the discovery of more examples of similar sets of genes, and detailed characterization of the biochemical pathways involved.

## Materials and methods

We set up a local basic local alignment search tool (BLAST) database of the proteins encoded in 26 completely sequenced fungal genomes (*A. niger*, *A. nidulans*, *A. terreus*, *A. flavus*, *A. oryzae*, *A. clavatus*, *N. fischeri*, *A. fumigatus Af293*, *A. fumigatus CEA10*, *C. immitis*, *C. posadasii*, *P. chrysogenum*, *U. reesii*, *S. sclerotiorum, F. graminearum*, *F. oxysporum*, *F. verticillioides*, *M. grisea*, *N. crassa*, *C. globosum*, *H. jecorina *(*T. reesei*), *N. haematococca *(*F. solani*), *P. chrysosporium*, *S. nodorum *(*P. nodorum*), C.*neoformans*, *U. maydis*). To find candidate *ACE1*-like clusters in other fungi, we used a two-step process outlined below.

In the first step, each protein encoded by the *M. grisea ACE1 *cluster was used as a query in protein-protein BLAST (BLASTP) searches against this database, and for each query the top 25 hits were retained provided that their E-values were less than 1e-4. Each set of proteins was aligned using ClustalW [49] and poorly aligned regions were removed using Gblocks [50]. Sequence alignments are available as Additional data file 2. Maximum likelihood trees were constructed using PHYML [51] with the JTT amino acid substitution matrix and four categories of substitution rates. Bootstrapping was done using the default options in PHYML with 100 replicates per run. To avoid long branch attraction problems we withdrew highly divergent sequences and repeated the alignment and tree reconstruction steps on the new sets. We also verified at each step that the alignment obtained after running Gblocks represented at least 30% of the initial protein sequence. Genes were considered as orthologs of an *M. grisea ACE1 *cluster gene if they grouped in a monophyletic clade with a bootstrap support of ≥70%.

Many of the genes identified in this first step were located in gene clusters. For each cluster so identified (defined as the presence of at least two homologs of *M. grisea ACE1 *cluster genes adjacent to one another) we then made a second step of analysis, examining any other genes that are physically located within these clusters but which were not picked up at the first step (either because their BLASTP E-values were too weak, or because they were not in the top 25 hits when the database was searched). This process added genes CHG05286.1, CHG05287.1, SNU00307.1 and FVEG_12610 to the analyses.

## Abbreviations

BLAST, basic local alignment search tool; HGT, horizontal gene transfer; LRT, likelihood ratio test.

## Authors' contributions

JC and MHL isolated the *M. grisea ACE1 *cluster and identified initial evidence of HGT. NK and JC conducted genome searches and phylogenetic analyses. KHW drew the figures. All authors contributed to writing the manuscript. All authors read and approved the final manuscript.

## Additional data files

The following additional data are available with the online version of this article: a figure (Additional data file 1) showing maximum likelihood trees for the *ACE1 *cluster genes that are not included in Figure 2 (*OXR1*, *BC2*, *OXR2*, *MFS1 *and *OME1*), and a data file (Additional data file 2) containing the sequence alignments used to produce Figure 2 and Additional data file 1.
